# Hippocampal connectivity in the aftermath of acute social stress

**DOI:** 10.1016/j.ynstr.2019.100195

**Published:** 2019-09-16

**Authors:** Jingjing Chang, Rongjun Yu

**Affiliations:** a Guangdong Key Laboratory of Mental Health and Cognitive Science, Center for Studies of Psychological Application, School of Psychology, South China Normal University, Guangzhou, China; b Department of Psychology, National University of Singapore, Singapore

**Keywords:** Stress, Hippocampus, Granger causal analysis, Insula, Midbrain, Thalamus

## Abstract

The hippocampus is a core brain region that responds to stress. Previous studies have found a dysconnectivity between hippocampus and other brain regions under acute and chronic stress. However, whether and how acute social stress influences the directed connectivity patterns from and to the hippocampus remains unclear. In this study, using a within-subject design and Granger causal analysis (GCA), we investigated the alterations of resting state effective connectivity from and to hippocampal subregions after an acute social stressor (the Trier Social Stress Test). Participants were engaged in stress and control conditions spaced approximately one month apart. Our findings showed that stress altered the information flows in the thalamus-hippocampus-insula/midbrain circuit. The changes in this circuit could also predict with high accuracy the stress and control conditions at the subject level. These hippocampus-related brain networks have been documented to be involved in emotional information processing and storage, as well as habitual responses. We speculate that alterations of the effective connectivity between these brain regions may be associated with the registering and encoding of threatening stimuli under stress. Our investigation of hippocampal functional connectivity at a subregional level may help elucidate the functional neurobiology of stress-related psychiatric disorders.

## 1 Introduction

Stress is a ubiquitous feature of fast-changing societies, and empirical studies have shown that stress can have a profound impact on various facets of emotional and cognitive functions (Sandi, 2013; Vogel et al., 2016). In extreme circumstances, stress can lead to psychogenic diseases such as major depressive disorder (MDD) or post-traumatic stress disorder (PTSD). The hypothalamo-pituitary-adrenal (HPA) axis plays an important role in response to threatening stimuli (stressors) by releasing glucocorticoids (cortisol in humans, and corticosterone in rodents) (J. P. Herman and Cullinan, 1997; Kalsbeek et al., 2012). The glucocorticoids play an important role in promoting survival by redistributing energy to critical functions in the face of stressors. However, the glucocorticoids need tight control to protect the individual from the harm of experiencing long-term alteration of homeostasis (J. Herman, McKlveen, Solomon, Carvalho-Netto and Myers, 2012; Tasker and Herman, 2011; Ulrich-Lai and Herman, 2009). The negative feedback inhibition of glucocorticoid could be mediated by mineralocorticoid receptors (MR) or glucocorticoid receptors (GR).

The hippocampus has a high density of both MR and GR, and is documented to be involved in glucocorticoid feedback inhibition (de Kloet et al., 2005; Sandi, 2013). Previous studies found that stimulating the hippocampus results in the decrease of glucocorticoid level (J. P. Herman et al., 2003), while lesion in this brain region causes prolonged HPA axis responses to stressors (J. Herman, Dolgas and Carlson, 1998). Using the Montreal Imaging Stress Task (MIST) to induce acute stress, Pruessner et al. found that the hippocampus volume was negatively correlated with the cortisol response to the stressor (Pruessner et al., 2005). It has been shown that acute and chronic stress also reduces synaptic strength, suppress neuronal propagation, induce morphological and functional changes in hippocampus (Diamond et al., 2007; L. Schwabe and Wolf, 2012).

Besides the effects of stress on the hippocampus itself, the temporal correlations between the hippocampus and other brain structures are also disrupted by acute and chronic stressors. For example, after stress was induced in a serial subtraction task, the connectivity between the hippocampus and amygdala was increased for up to 2 h (Vaisvaser et al., 2013). In an appetitive conditioning task, it was found that compared to controls, stressed participants exhibited enhanced functional connectivity between the hippocampus and three other regions, namely amygdala, ventral anterior cingulate cortex, and orbitofrontal cortex (Kruse et al., 2017). The functional connectivity (FC) analysis describes the dependencies between two or more brain regions without any assumption about the direction of these correlations (Seth et al., 2015). In contrast, effective connectivity can be used to explore the direction of functional interaction among brain regions.

The hippocampal structure is comprised of functionally heterogeneous subfields: the cornu ammonis (CA1-CA3), dentate gyrus (DG), and subicular complex (Subc) (Amunts et al., 2005; Jones and McHugh, 2011). In addition to studies documenting the effects of stress on the whole hippocampus (L. Schwabe and Wolf, 2012; Vaisvaser et al., 2013), other animal and human studies have demonstrated the effects of stress on subfields of the hippocampus. For instance, a recent study found that the central stress response indicated by c-Fos in male rats only decreased in the CA1 subfield of the hippocampus after injection of MR/GR modulator (Nguyen et al., 2017). Another study comparing MDD patients and controls also found evidence of regional specificity (Travis et al., 2016). Eight hours after awakening, the salivary cortisol in MDD patients was higher than in controls and the increases were negatively correlated with the left CA1-3 and left hippocampal head volume, while, in healthy controls, the mean cortisol level was negatively correlated with right CA1-3 and right hippocampal head volume.

Although previous studies have found stress-induced structural and functional alterations in the hippocampus and its subregions, whether and how stress influences the effective connectivity patterns involving the hippocampal subfields remains unclear. Compared with exploring changes in brain region activation or alterations in undirectional functional connectivity, effective connectivity analysis could provide more information about how distributed neural systems influence each other (K. J. Friston, 2011). In the present study, using a within-subject design and Granger causality analysis (GCA), we investigated how effective connectivity from and to hippocampal subregions is altered after acute psychological social stress. Compared with the between-subject design adopted by previous studies (Kruse et al., 2017; Maier et al., 2015), a within-subject design reduces error variance associated with individual differences between groups. GCA is a powerful method to examine brain information flow between brain regions, relying on a simple idea that a brain region X Granger causes the other brain region Y if time courses of X precede and are useful to predict the time series of Y (Granger, 1969; Guo et al., 2015a, Guo et al., 2015b; Seth et al., 2015; Zhang et al., 2017). Another widely used method to explore effective connectivity is dynamic causal modeling (DCM). DCM is a hypothesis-driven approach and requires prespecified models to decide which one fits the observed data best using Bayesian frameworks (K. J. Friston, Harrison and Penny, 2003). GCA is a data-driven approach and requires fewer parameters than the DCM (Seth et al., 2015). GCA has been widely used in patient and healthy population studies (Bellucci et al., 2017; Guo et al., 2015a, Guo et al., 2015b; Hamilton et al., 2011). For example, Hamilton et al. adopted both bivariate and multivariate GCA to identify the brain structures whose activity had causal connectivity (e.g., following or preceding) with the activation in ventral anterior cingulate cortex in MDD, thus providing more information about the neural mechanisms underlying this disorder (J. P. Hamilton et al., 2011a, Hamilton et al., 2011b).

Dysregulation of emotions is a common pathophysiological feature of stress-related disorders (Seligowski et al., 2015; Tobia et al., 2017). In the present study, we conducted a whole-brain analysis to explore the stress-induced effective connectivity alteration of hippocampal subregions with brain areas restricted to the limbic system, including the bilateral amygdala, anterior cingulate cortex, midbrain, insula and thalamus. These brain structures are structurally and functionally connected with the hippocampus, and constitute a neural network associated with the processing and modulation of emotions, and are all documented to be vulnerable to acute and chronic stress (Arnold Anteraper et al., 2014; X. Cao et al., 2012a, Cao et al., 2012b; A. David and Pierre, 2009; Phillips et al., 2003; Price and Drevets, 2010). We hypothesized that in comparison with a non-stressful condition, there could be stronger effective connectivity between the hippocampus and these brain regions of the limbic system to cope with emotional information induced by a stress condition.

As the significant differences between “no-stress” and “stress” conditions in hippocampal subregions-related effective connectivity were based on statistical separation (e.g., the paired-t-test), the predictive power of these neural signatures remains unclear (Deshpande et al., 2010a, Deshpande et al., 2010b; D Rangaprakash et al., 2017a, Rangaprakash et al., 2017b). Hence we further explored whether this significantly different information flows from and to hippocampal subregions could be used to distinguish brain states in stress and control conditions. We employed the support vector machine (SVM), a machine learning algorithm that can build a model to optimally categorize data correctly after training (Cortes and Vapnik, 1995). SVM is popular for its high flexibility and accuracy as well as its capability to deal with numerous features with few training patterns (Guyon et al., 2002; Liu et al., 2017; Lou et al., 2017).

## 2 Materials and methods

### 2.1 Participants

Participants, who were recruited from the local community, included 30 healthy right-handed volunteers (15 females) whose ages ranged from 18 to 25 years old (*M* = 20.6, *SD* = 2.0). None reported previous participation in a stress-related research study. None of them reported any history of major medical, psychiatric, or neurological diseases. All participants provided written informed consent according to protocols approved by the South China Normal University Institutional Review Board. Before the experiment, participants were instructed to refrain from intense exercise and caffeine 12 h before the study.

### 2.2 Experimental design

**Fig. 1:**
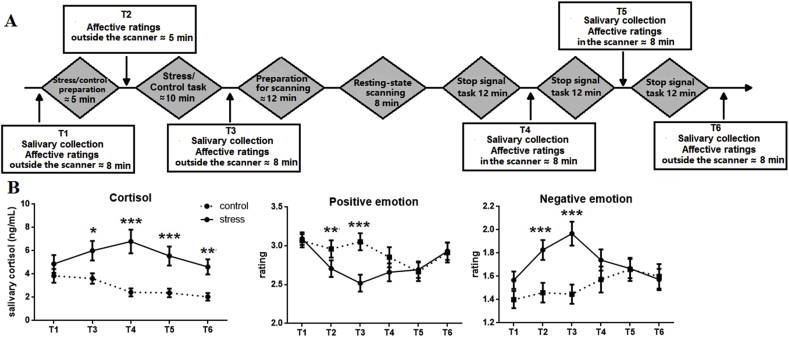
Experimental procedure and manipulation check. (A) The timeline of the experiment. After an acclimation period of 20 min following arrival, participants were asked to go through the Trier Social Stress Test (TSST) with (stress condition) or without (control condition) social evaluative processes. After the formal tasks, resting state fMRI data were collected. Saliva samples were collected at T1, T3, T4, T5, and T6. Affective ratings were collected at T1, T2, T3, T4, T5, and T6. (B) Cortisol and positive/negative emotional responses under control and stress conditions (mean and standard error). *p < 0.05, **p < 0.01, ***p < 0.001.

As shown in Fig. 1A, after an acclimation period of 20 min following arrival, baseline saliva samples and affect ratings (see *physiological and psychological measures)* were collected (T1). Participants were then instructed to complete either the stress or control task (see *stress induction*) and given 5 min to prepare, after which affect ratings were again recorded (T2). Participants then completed the stress or control task, with saliva samples and affect ratings collected upon task completion (T3). Next, 8 min of resting-state fMRI data were collected. After the scan, participants completed three sessions of the stop signal task (SST) (Hu et al., 2016), the results of which are reported elsewhere. After each session of SST, saliva samples and affect ratings were collected (T4, T5, and T6). Participants were exposed to the acute stress and control conditions in two separate sessions with at least a 30-day interval between the two sessions. Participants were randomly assigned to two groups based on condition order, such that half completed the stress condition first and the other half completed the control condition first. Between-group analysis showed that the two groups did not significantly differ in age or gender (*p*s > 0.5), indicating that the randomly assigned two groups were comparable. Following previous studies (Qin et al., 2012; L. Schwabe and Wolf, 2012), all sessions were conducted in the afternoon (i.e., between 1 p.m. and 6 p.m.), as levels of endogenous cortisol in the afternoon have been reported to be relatively stable and low (Deuschle et al., 1997).

### 2.3 Stress induction

Participants completed the Trier Social Stress Test (TSST), a well-validated stressor (Kirschbaum et al., 1993). The TSST included a preparation period (5 min) and a formal task period (5-min public speaking task and 5-min mental arithmetic task). In the stress condition, participants were instructed to prepare a job application and introduce themselves in front of a committee and a video camera. To increase task engagement, participants were asked to write down their dream job before the preparation period. They were instructed to convince the committee that they were the most suitable candidate for this position. The committee members (one woman and one man) were trained to remain emotionally neutral during the speech. Upon completion of the speech, participants were asked to subtract the number 13 serially from 1022 and report their calculations in English as quickly and accurately as possible for 5 min. If they committed any error, they were asked to restart from 1022. Having participants respond in a foreign language (i.e., English) was expected to increase the difficulty of the mental arithmetic task for the participants and possibly further increase their stress level. Participants used Chinese in all other parts of the experiment.

In the control condition, to ensure a comparable cognitive load, participants went through the same tasks in an empty room without the committee and video camera. In other words, there was no social evaluative stress in the control condition.

### 2.4 Physiological and psychological measures

To ascertain whether the manipulation of acute stress was effective in terms of changes in physiological and psychological responses, salivary cortisol and affect ratings were assessed at multiple time points throughout the experiment (see Fig. 1A). Saliva samples were collected using Salivettes (Sarstedt, Germany) and were stored at −15 °C until assayed. Cortisol concentrations in saliva (in ng/mL) were measured by performing ELISA (catalog No. SLV 4635; DRG, Germany).

Positive and negative affect were measured using the Positive Affect and Negative Affect Schedule (PANAS), and this measure has shown high reliability in adults samples (Van Marle, Hermans, Qin and Fernández, 2010; Watson et al., 1988). Positive emotions included calm, relaxed, peaceful, confident, and energetic; negative emotions included nervous, anxious, scared, tired, and upset. Participants rated items on a four-point scale from 1 (“not at all”) to 4 (“extremely”).

### 2.5 Image data acquisition

Brain images were obtained with a 3-T MRI scanner (Siemens) at the Brain Imaging Center at South China Normal University. T1-weighted images were acquired with the following parameters: repetition time = 1900 ms, echo time = 2.52 ms, field of view = 256 × 256 mm^2^, flip angle = 9°, matrix size = 256 × 256, and 1 mm^3^ isotropic voxel. T2*-weighted echo-planar images (EPI) were obtained with blood oxygenation level-dependent (BOLD) contrast. Thirty-two axial slices covering the whole brain were acquired with TR = 2000 ms, TE = 25 ms, flip angle = 85°, field of view = 220 × 220 mm, matrix size = 64 × 64, in-plane voxel size = 3 × 3 mm, and slice thickness = 4 mm with no gap. Slice scanning order was ascending interleaved. A total of 240 images were acquired for the resting state scan. During the resting state scanning, all participants were requested to close their eyes.

### 2.6 Imaging preprocessing

The fMRI data were preprocessed and analyzed using Statistical Parametric Mapping version 8 (SPM8, Wellcome Department of Imaging Neuroscience, University College London, U.K.) and Data Processing & Analysis for (Resting-State) Brain Imaging (DPABI; http://rfmri.org/DPABI) (Yan et al., 2016). After discarding the first 10 vol, the remaining 230 fMRI volumes were first slice-time corrected and later head-motion corrected using a least squares approach and a 24-parameter autoregressive model (Friston 24-parameter model) (K. J. Friston, Williams, Howard, Frackowiak and Turner, 1996). The 24 parameters included six head motion parameters, six head motion parameters one-time point before, and the 12 corresponding squared items. No participant's head motion exceeded 2.0 mm in translation or 2° in rotation. We further calculated frame-wise displacement (FD), which indexes volume-to-volume changes in head position (Power et al., 2014). The mean FD in stress and control groups were 0.11 ± 0.03 and 0.12 ± 0.03, respectively. One-sample *t*-test showed that they were significantly less than 0.2 mm (both *p* < 0.001). Furthermore, the paired *t*-test showed no significant differences in FD between the two conditions (*p* = 0.43).

Subsequently, T1-weighted and functional images were reoriented by hand to optimize alignment for co-registration, segmentation, and normalization (N. Wang et al., 2017). Individual T1-weighted images were co-registered to the mean motion-corrected functional image. The resulting aligned images were then segmented into gray matter, white matter, and cerebrospinal fluid (CSF). To remove the nuisance signal, the 24 head-motion parameters, CSF, and white matter were regressed out. Next, the segmented images were transformed into Montreal Neurological Institute (MNI) space via the Diffeomorphic Anatomical Registration Through Exponentiated Lie Algebra (DARTEL) technique and used to generate a study-specific template (Ashburner, 2007). The functional images were normalized into a standardized MNI space using the specific template, re-sampled to a 3 mm × 3 mm × 4 mm voxel, spatially smoothed with a 6 mm FWHM Gaussian filter, and temporally band-pass filtered into 0.01–0.1 Hz to reduce the effect of very low-frequency drift and high-frequency physiological noise.

### 2.7 Granger causal analysis (GCA)

#### 2.7.1 Definition of the seed region of interest (ROI)

**Fig. 2:**
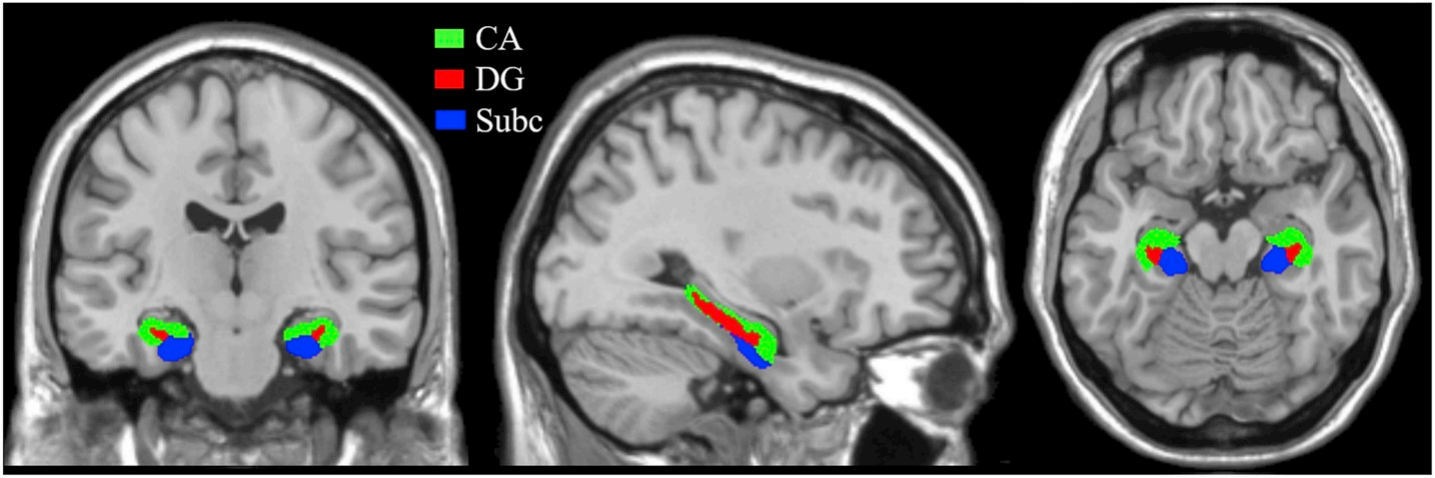
Locations of hippocampal subregions. Green: CA (CA1-CA3 combined); Red: DG (fascia dentata and CA4); Blue: Subc. (For interpretation of the references to colour in this figure legend, the reader is referred to the Web version of this article.)

To be consistent with previous studies (Atienza et al., 2011; Kurth et al., 2017; Z. Wang et al., 2015), we used the maximum probabilistic map of three hippocampal subregions in both the left and right hemispheres derived from SPM Anatomy Toolbox v2.2b (Amunts et al., 2005). These 3 subregions were located in the CA (including CA1, CA2 and CA3), DG and Subc (see Fig. 2). Only voxels with at least a 50% probability of belonging in one of these subregions were included in an ROI, and each voxel was assigned to only one subregion. Subregion maps for the two hemispheres were combined to create bilateral ROIs for the CA, DG, and Subc subregions.

#### 2.7.2 GCA processing

We calculated the voxel-wise bivariate coefficient GCA by using the REST-GCA in the REST toolbox (http://www.restfmri.net; (Song et al., 2011). We estimated the Granger causal effects between the reference time series of the seed regions (bilateral CA, DG, and Subc) and the time series of each voxel within the whole brain. Two analyses were conducted to explore voxel-wise GCA: seed-to-whole-brain and whole-brain-to-seed. The seed-to-whole-brain analysis explored the driving or inhibitory effects of seeds on other voxels in the brain, whereas the whole-brain-to-seed analysis explored the excitatory or depressive effects of other voxels on the seeds (J. P. Hamilton et al., 2011a, Hamilton et al., 2011b; Ji et al., 2013). Vector autoregressive models were used to estimate Granger causality to determine whether or not the past value of a time series could correctly forecast the present value of another. If the combination of the past values of the time series X and Y could estimate the current value of Y more accurately than the past value of Y alone, then the time series X is said to have a causal effect on time series Y (Zang et al., 2012). This is to say that if the signed path coefficient is significantly different from zero in the stress or control condition, then it is said that X shows significant Granger prediction on Y (Chen et al., 2009; J Paul Hamilton et al., 2011). In particular, positive signed path coefficients (i.e., those significantly larger than zero) indicate that increases in X could predict the current increases in Y, whereas negative signed path coefficients (i.e., those significantly smaller than zero) indicate that increases in X in the last time point could predict current decreases in Y. Further, because signed path coefficients are considered to be normally distributed, parametric statistical analyses can be used to make group-level inferences (Zang et al., 2012).

The bivariate voxel-wise GCA maps of each seed ROI for each condition were fed to a flexible factorial analysis with the following factors: Subject, Condition (stress vs. control), and Subregion (bilateral CA/DG/Subc). Within this ANOVA we calculated both main effects (condition and subregion) as well as the condition × subregion interaction. To further visualize significant interaction effects, the average Granger causality values in significant brain regions were extracted and subjected to post-hoc tests. For reported flexible factorial analyses, an uncorrected voxel threshold of *p* < 0.005 followed by a family-wise error (FWE) corrected threshold of *p* < 0.05 using small volume correction (SVC) was set. The ROIs for SVC included the bilateral amygdala, anterior cingulate cortex, midbrain, insula and thalamus. The ROIs were defined using the corresponding AAL mask(Tzourio-Mazoyer et al., 2007). These brain regions are structurally and functionally connected to the hippocampus and constitute a neural network of emotion processing and modulation (Xiaohua Cao et al., 2012; A. David and Pierre, 2009; Price and Drevets, 2010).

### 2.8 Classification analysis using SVM

#### 2.8.1 SVM processing

To further estimate the accuracy of using GCA values to predict stress or control state, we adopted the SVM method. The SVM was conducted using the LIBSVM software package (http://www.csie.ntu.edu.tw/∼cjlin/libsvm/) (C.-C. Chang and Lin, 2011). SVM requires a training dataset to learn differences between different conditions and a test dataset to evaluate classification performance on unobserved data. Our data were trained by providing label pairs (x_i_, c_i_), i = 1, …, l, where x_i_ ∈ R^n^. x_i_ represents the average Granger causality values from brain structures that significantly different between stress and control conditions, and c corresponds to the class label. In our sample, the stress and control conditions were assigned class labels “c = +1” and “c = −1”, respectively. These Granger causality values in training dataset and test dataset were normalized respectively, i.e. converted to z scores.

#### 2.8.2 Feature ranking

Before training the classifiers, the SVM–Ranking Feature Extraction (SVM–RFE) algorithm was used to rank the features (i.e., the significantly different Granger causal values between stress and control conditions (see Fig. 4) according to their potential for discriminating between stress and control conditions (Guyon et al., 2002; Lou et al., 2017). SVM–RFE returned a ranking of the classification features (see Table 1) by training SVM with a linear kernel and removing the feature with the smallest ranking criterion. The SVM–RFE feature ranking function was conducted using the LIBSVM software package.

**Table 1:** The rank of features.

Ranking	Features
1	average Granger causality values from CA to left insula
2	average Granger causality values from CA to left midbrain
3	average Granger causality values from DG to left insula
4	average Granger causality values from right thalamus to DG
5	average Granger causality values from left thalamus to DG
6	average Granger causality values from left thalamus to CA
7	average Granger causality values from left thalamus to Subc

CA,cornu ammonis; DG, dentate gyrus; Subc, subicular complex.

#### 2.8.3 SVM models evaluation and selection

After feature ranking, we firstly used the best feature to train the classifiers. Then, we performed new tests by including each feature one-by-one according to its potential for discriminating between two classes of interest (i.e., its rank). For each combination of features, the classifier was trained and then applied to classify the validation samples. The LIBSVM classifier algorithm was applied using leave-one-subject-per-group-out cross-validation (LOSPGOCV) technique, which is appropriate for a within-subject design we used (see Pattern Recognition for Neuroimaging Toolbox manual 10.5.1) (Schrouff et al., 2013). Specifically, in the present study, two samples, which were the Granger causality values of the same participant under stress and control conditions, were selected as the testing dataset in each LOSPGOCV procedure. Then the classification model was constructed in the training stage with the remaining 58 Granger causality values (i.e., the other 29 participants' data under stress and control condition) provided as features and their condition labels as output. After that, this model was used in the prediction stage to predict the selected testing samples' condition. By repeatedly leaving each participant's data out as the test set, we obtained the average classification rate from 30 leave-one-subject-per-group-out procedures (Fan et al., 2005). To determine whether the obtained mean accuracy was significantly higher than chance, we applied permutation test. We permuted the labels (control or stress) randomly across the entire sample 1,000 times, and reapplied the entire classification procedure each time (Cui et al., 2016). The *P* value was calculated by dividing the number of permutations which had a higher classification rate than the real dataset by 1000 (i.e., the total number of permutation).

Three aspects of the SVM models’ performance were evaluated: (1) mean classification accuracy of 30 LOSPGOCV procedures (i.e., the mean fraction of correctly classified condition out of two conditions of a participant in the test set), (2) sensitivity (i.e., the ratio of correctly classified participants in the stress condition to the total number of participants in the stress condition in the test set), and (3) specificity (i.e., the ratio of correctly classified participants in the control condition to the total number of participants in the control condition in the test set; (Akay, 2009; Lou et al., 2017).

## 3 Results

### 3.1 Physiological responses to acute stress

For all reported analyses, Greenhouse-Geisser correction was applied when the assumption of sphericity was violated. To check whether the manipulation of acute stress was effective, we carried out a Treatment (control vs. stress) × Time Point (T1, T3, T4, T5, and T6) repeated-measures ANOVA on cortisol level (see Fig. 1B). Three participants’ cortisol samples could not be assayed due to insufficient saliva, leaving data from 27 participants for analysis. Results showed significant main effects of treatment (*F*(1, 26) = 16.258, *p* < 0.001, *η*^*2*^ = 0.385) and time point (*F*(4, 104) = 4.159, *p* = 0.01, *η*^*2*^ = 0.138). The interaction between treatment and time point was also significant (*F*(4, 104) = 4.453, *p* = 0.006, *η*^*2*^ = 0.146). Being consistent with previous studies (Dickerson and Kemeny, 2004; Ginis et al., 2012), which suggest that the cortisol reactivity reaches its peak between 21 and 40 min following stressor onset, post-hoc *t* tests showed that the stress condition induced higher cortisol than the control condition at T3 (*t*(26) = 2.677, *p* = 0.013), T4 (*t*(26) = 4.503, *p* < 0.001), T5 (*t*(26) = 4.112, *p* < 0.001), and T6 (*t*(26) = 3.947, *p* = 0.001).

For positive and negative emotion ratings, we also carried out two Treatment (control vs. stress) × Time Point (T1, T2, T3, T4, T5, and T6) repeated-measures ANOVAs on positive emotion ratings and on negative emotion ratings (see Fig. 1B). For positive emotion ratings, results showed significant main effects of treatment (*F*(1, 29) = 4.355, *p* = 0.046, *η*^*2*^ = 0.131) and time point (*F*(5, 145) = 6.556, *p* < 0.001, *η*^*2*^ = 0.184). The interaction between treatment and time point was also significant (*F*(5, 145) = 7.505, *p* < 0.001, *η*^*2*^ = 0.206). Post-hoc *t* tests showed significantly lower positive emotion ratings in the stress condition than in the control condition at T2 (*t*(29) = −2.801, *p* = 0.009) and T3 (*t*(29) = −4.817, *p* < 0.001). For negative emotion ratings, results showed significant main effects of treatment (*F*(1, 29) = 8.553, *p* = 0.007, *η*^*2*^ = 0.228) and time point (*F*(5, 145) = 3.354, *p* = 0.015, *η*^*2*^ = 0.104). The interaction between treatment and time point was also significant (*F*(5, 145) = 7.495, *p* < 0.001, *η*^*2*^ = 0.205). Post-hoc *t* tests showed significantly higher negative emotion ratings in the stress condition than in the control condition at T2 (*t*(29) = 4.413, *p* < 0.001) and T3 (*t*(29) = 4.763, *p* < 0.001). Taken together, these findings suggest that acute stress increased cortisol level and modulated emotional experience.

### 3.2 GCA results

#### 3.2.1 Seed-to-whole-brain analysis

We first carried out a flexible factorial analysis with the following factors: Subject, Condition (stress vs. control), and Subregion (bilateral CA/DG/Subc). Results revealed a significant main effect of condition, with increased driving effects from the seed regions to the right thalamus ([21–27 4], voxel = 29, *p* = 0.037, SVC), and left thalamus ([-18 -24 0], voxel = 60, *p* < 0.001, SVC) in the control minus stress comparison. There was a significant effect in the left insula ([-45 15 -12], voxel = 3, *p* = 0.025, SVC) for the stress minus control comparison, showing that the seed regions had increased driving effect to left insula in the stress condition than in the control condition. Consistent with previous studies, the main effect of subregion is situated within broader brain regions such as the bilateral cingulate gyrus, occipital lobe, parahippocampal gyrus, orbital cortex, prefrontal cortex, cerebellum, and temporal lobe, which are structurally and functionally connected with the hippocampus (Blankenship et al., 2017).

**Fig. 3:**
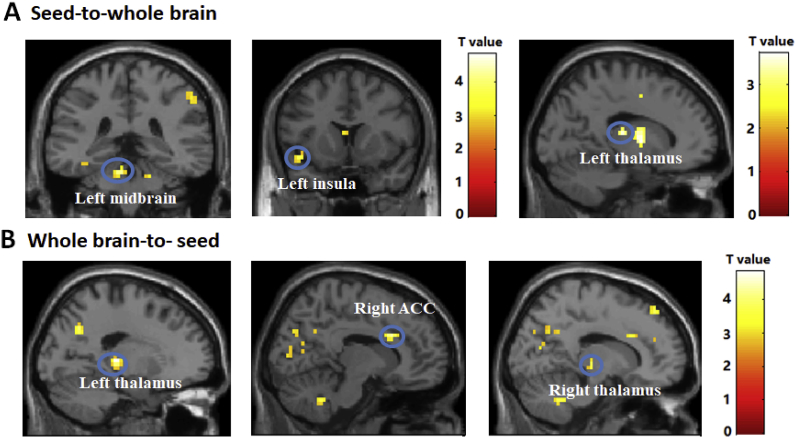
(A) Seed-to-Whole-Brain: brain clusters that showed significant Condition (stress vs. control) × Subregion (bilateral CA/DG/Subc) interaction in flexible factorial analysis; (B) Whole-Brain-to-Seed: brain clusters that showed significant Condition (stress vs. control) × Subregion (bilateral CA/DG/Subc) interaction in flexible factorial analysis. ACC, anterior cingulate cortex. *p* < 0.05 (SVC corrected).

**Fig. 4:**
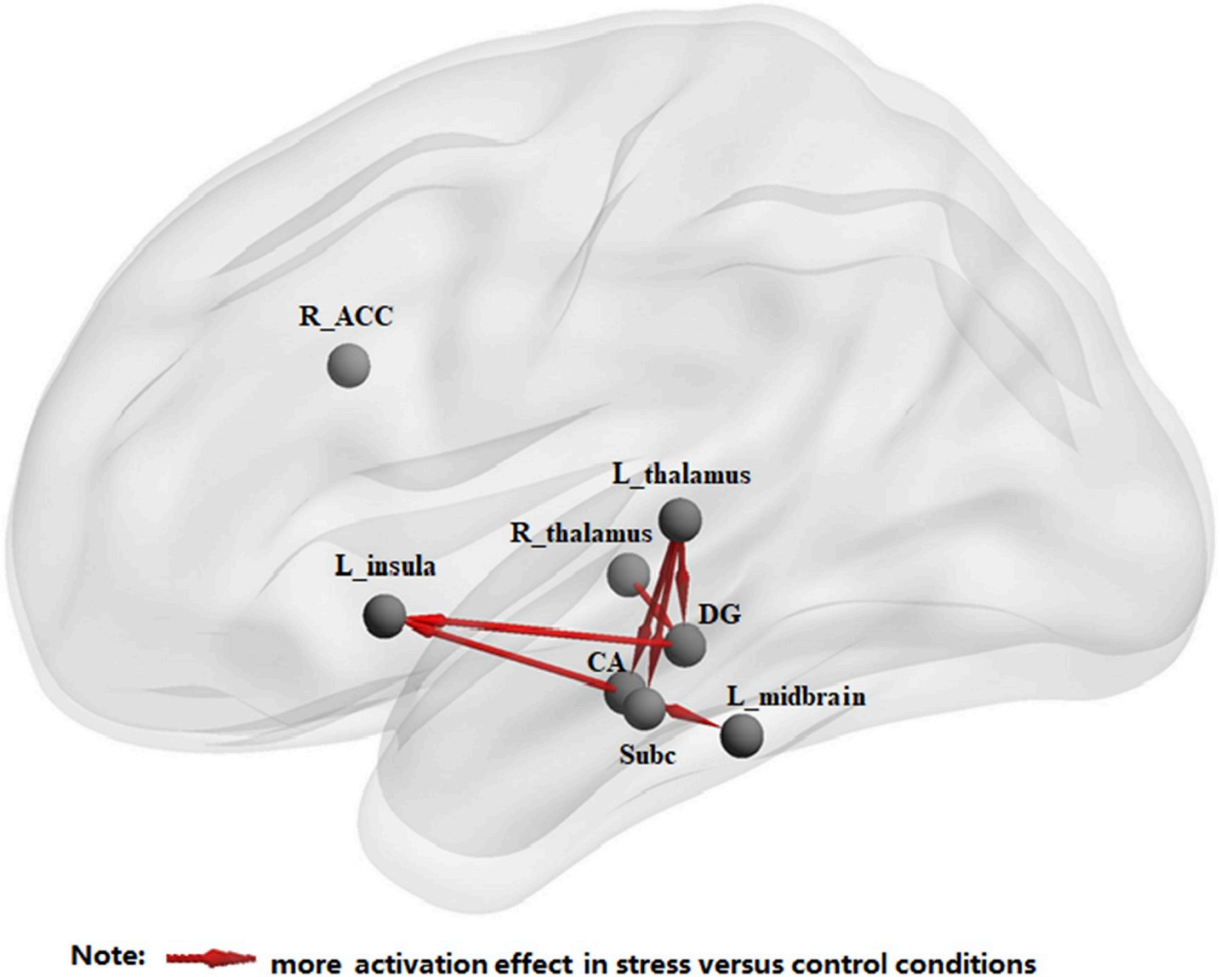
A statistical map of a between-condition comparison of path coefficients from bivariate GCA, including hippocampal subregions, left midbrain, left thalamus, left insula, right thalamus and right anterior cingulate cortex (all *p* values < 0.05, uncorrected). CA, cornu ammonis; DG, dentate gyrus; Subc, subicular complex; L, left; R, right; ACC, anterior cingulate cortex.

The interaction between condition (stress vs. control) and subregion (bilateral CA/DG/Subc) revealed significant clusters in the left insula ([-45 15 -12], voxel = 10, *p* = 0.047, SVC), left midbrain ([-6 -36 -24], voxel = 5, *p* = 0.005, SVC), and left thalamus ([-15 -21 12], voxel = 8, *p* = 0.041, SVC; see Fig. 3A). The average Granger causality values from these significant clusters were extracted and submitted to a post-hoc test. The results showed that increased activity in the CA and DG predicted subsequent increases in activation of the left insula to a greater extent in the stress condition than in the control condition (*t* = 3.846, *p* = 0.001; *t* = 3.180, *p* = 0.003). Further, we found increased activity in the CA to be predictive of higher activity in the left midbrain in the stress condition (*t* = 3.105, *p* = 0.004). Finally, compared with the control condition, the increased activity in the CA and DG predicted lower subsequent increases in left thalamus activity in the stress condition (*t* = −3.362, *p* = 0.002; *t* = −2.571, *p* = 0.016) (see Table 2). Being consistent with previous studies, we reported the Granger causality values which were not only significantly different between the stress and control conditions, but also showed a within-condition effect in the stress condition (i.e., the signed path coefficients in the stress condition were significantly different from zero) in Fig. 4 (J. P. Hamilton et al., 2011a, Hamilton et al., 2011b).

**Table 2:** The mean path coefficients.

Seed-to-whole-brain			Whole-brain-to-seed			
	L_insula	L_midbrain	L_thalamus	R_ACC	L_thalamus	R_thalamus
**Stress-control**						
**CA**	**0.301**	**0.168**	**−0.151**	- 0.005	**0.026**	0.015
**DG**	**0.292**	0.090	**−0.122**	0.001	**0.053**	**0.037**
**Subc**	0.101	−0.003	−0.048	**0.038**	**0.080**	**0.055**
**Stress**						
**CA**	**0.167**	**0.144**	0.020	**- 0.025**	**0.015**	0.001
**DG**	**0.196**	**0.112**	0.036	**- 0.021**	**0.024**	**0.014**
**Subc**	0.040	0.001	**0.074**	−0.004	**0.024**	0.009
**Control**						
**CA**	**−0.133**	−0.024	**0.171**	**−0.020**	−0.011	−0.013
**DG**	−0.095	0.022	**0.158**	**−0.022**	**−0.029**	**−0.023**
**Subc**	−0.061	0.003	**0.122**	**−0.042**	**−0.056**	**−0.046**

Note: The mean path coefficients in bold are significantly different from zero.

CA, cornu ammonis; DG, dentate gyrus; Subc, subicular complex; L, left; R, right; ACC, anterior cingulate cortex.

#### 3.2.2 Whole-brain-to-seed analysis

We carried out a flexible factorial analysis with the following factors: Subject, Condition (stress vs. control), and Subregion (bilateral CA/DG/Subc). Results showed a significant main effect of condition in the left and right thalamus. Specifically, the stress condition minus the control condition revealed that activity in the left and right thalamus predicted subsequent increases in the activity of hippocampal subregions to a significantly greater extent in the stress condition than in the control condition ([-18 -24 4], voxel = 31, *p* = 0.003; [21–27 4], voxel = 16, *p* = 0.036). No significant difference in the brain regions of interest were found for the control minus stress comparison. Consistent with the reported results of the seed-to-whole-brain analysis, the whole-brain-to-seed analysis revealed a main effect of the subregion in bilateral parietooccipital regions, amygdala, putamen, caudate, cingulate gyrus, parahippocampal gyrus, and other regions (Blankenship et al., 2017; D. Rangaprakash et al., 2017a, Rangaprakash et al., 2017b).

The interaction between condition (stress vs. control) and subregion (bilateral CA/DG/Subc) revealed significant clusters in the right anterior cingulate cortex (ACC; [12 18 28], voxel = 16, *p* = 0.01, SVC), left thalamus ([-18 -27 4], voxel = 20, *p* = 0.001, SVC), and right thalamus ([15–21 0], voxel = 8, *p* = 0.032, SVC; see Fig. 3B). The average Granger causality values extracted from these significant clusters were submitted to a post-hoc test. The results of post-hoc *t*-test showed that increased activity in the right ACC predicted subsequent increases in activation of the Subc to a greater extent in the stress condition than in the control condition (*t* = 2.427, *p* = 0.022). Increased activity in the left thalamus predicted subsequent increases in activation of the CA, DG, and Subc to a greater extent in the stress condition than in the control condition (*t* = 2.824, *p* = 0.008; *t* = 5.310, *p* < 0.001; *t* = 5.896, *p* < 0.001). Further, we found increased activity in the right thalamus to be predictive of higher activity in the DG and Subc in the stress condition (*t* = 3.195, *p* = 0.003; *t* = 3.808, *p* = 0.001) (see Table 2). For this whole-brain-to-seed analysis, we also reported the Granger causality values which were not only significantly different between the stress and control conditions, but also showed a within-condition effect in the stress condition in Fig. 4 (Hamilton et al., 2011a, Hamilton et al., 2011b).

#### 3.2.3 SVM results

The accuracy of the SVM models varied from 65.0% to 76.7%, depending on the combination of input features (see Table 3). The model that achieved the highest accuracy with the lowest number of features included Granger causality values from CA to left insula, from CA to left midbrain, from DG to left midbrain, and from right thalamus to DG. This model obtained an accuracy of 76.7%, a sensitivity of 70.0%, and a specificity of 83.3% (see Table 3). The permutation test showed that the accuracy was significantly higher that value expected by chance (*p* = 0.001), suggesting the model has remarkable predictive power.

**Table 3:** Accuracy, sensitivity, specificity, and Kappa index for the SVM model with different combinations of input features.

Number of features	Accuracy %	Sensitivity	Specificity	Kappa	*p*
1	65.000	0.767	0.533	0.700	0.009**
2	73.333	0.733	0.733	0.533	0.001**
3	70.000	0.700	0.700	0.600	0.002**
4	76.667	0.700	0.833	0.467	0.001**
5	76.667	0.733	0.800	0.467	0.001**
6	75.000	0.700	0.800	0.500	0.001**
7	73.333	0.733	0.733	0.533	0.001**

**permutation test, *p* < 0.01.

## 4 Discussion

We combined resting-state fMRI with GCA to investigate the effect of acute social stress on the effective connectivity between the hippocampal subregions (bilateral CA, DG, and Subc) and other brain regions. The SVM was adopted to further estimate the accuracy of using GCA values to predict stress and control state. The results showed that the effective connectivity between hippocampal subregions and brain regions related to emotion processing and modulation altered after acute stress exposure, including the insula, thalamus, and midbrain. Specifically, increased activity in the CA and DG predicted subsequent increases in activation of the left insula to a greater extent in the stress condition than in the control condition. Further, we found that increased activity in the CA predicted higher left midbrain activity in the stress condition (relative to the control condition). Compared with control condition, the increases in the left thalamus activity predicted subsequent increases in activation in all three hippocampal subregions (CA, DG, and Subc), and increased activity in the right thalamus predicted higher activity in the DG after stress exposure. Of these, the combination of four information flows, including from CA to left insula, from CA to left midbrain, from DG to left midbrain, and from right thalamus to DG, had high discriminative power of stress and control condition.

The Granger causality values from CA and DG to left insula were significantly different between stress and control conditions. Specifically, activity in the CA and DG predicted subsequent activation of the left insula to a greater extent in the stress than in the control condition. Previous works have shown that the insula is involved in processing of emotional information, especially aversive stimulations, such as pain and disgust (Geuter et al., 2017; Picó-Pérez et al., 2017; Uddin et al., 2017; Ying et al., 2018). Several lines of researches further explored the role of insula in stress-related disorders (Chu et al., 2018; Kandilarova et al., 2018). Using spectral dynamic causal modeling, Kandilarova et al. found that the main differences between depressed patients and healthy controls were the alteration of effective connectivity of insula with middle frontal gyrus and amygdala, showing a critical role of the insula in the mechanism of depression (Kandilarova et al., 2018). Recently, one study adopting a tractography algorithm with precise estimation found intense structural connectivity between insula and hippocampus (Ghaziri et al., 2018). In addition, compared with trustworthy facial expressions, the untrustworthy expressions induced more activation in insula, and the hippocampus activation was positively correlated with the insula activation when participants recognized the untrustworthy expressions with high confidence in retrieval stage, suggesting that the connectivity between hippocampus and insula supports negative signal processing and encoding (Tsukiura et al., 2012). In the present study, we adopted TSST in which social evaluation was used to make participants feel threatened and experience negative emotions. We speculated that the enhanced effective connectivity between hippocampal subregions and insula after acute stress exposure might be involved in the processing of negative social experience.

The current results show that increased activity in CA under stress predicts subsequent increase in activation in the left midbrain, including periaqueductal gray (PAG, [-6 -33 -20], voxel = 3, *p* = 0.013, SVC in a 6 mm sphere centered at MNI [-5, −32, −18]) (D. Mobbs et al., 2007). Previous studies showed that midbrain PAG triggered habitual defense responses, for example, flight, fight, or freeze, when the threat was imminent (Dean Mobbs, Hagan, Dalgleish, Silston and Prévost, 2015; D. Mobbs et al., 2009; D. Mobbs et al., 2007). For example, Dean et al. found that when participants anticipated an imminent high shock, the PAG was activated, resulting in panic reactions (D. Mobbs et al., 2007). These findings were consistent with previous findings that stress favors a habitual automated system over a cognitively demanding deliberative system (J. Chang and Yu, 2018a; Lars Schwabe and Wolf, 2013; Yu, 2016). The habitual behavior may improve response efficiency and thus be conductive to coping with current stress (Lars Schwabe and Wolf, 2011). Furthermore, midbrain PAG also responds to negative social emotion (Buhle et al., 2012; Linnman et al., 2012). For instance, a conjunction analysis showed that the midbrain PAG was activated by both phasic heat and negative emotion pictures, and these results were confirmed by 8 independent datasets (Buhle et al., 2012). In the present study, after acute stress exposure (TSST), participants’ emotion ratings showed that negative emotion significantly increased, and positive emotion significantly decreased. Thus the midbrain PAG might be involved in the negative emotion processing induced by our experimental manipulation. Further, our findings were consistent with previous studies showing that the connectivity between hippocampus and PAG might be related to the transition of temporary emotional experience to long-term memory (Egorova et al., 2015; Roy et al., 2014). For example, Egorova et al. found that hippocampus-PAG connectivity was significantly decreased after repeated verum acupuncture relative to sham acupuncture, representing the revaluation of the aversive pain state and update of nociceptive memory after verum acupuncture (Egorova et al., 2015). We speculate that the enhanced interaction of hippocampus and PAG after stress exposure might facilitate the transfer of negative emotional experiences into emotion memory so that the individual can better cope with future stressful events (Henckens et al., 2009). In addition, in the present study, we found that the altered effective connectivity from hippocampal cortex to other brain regions was focused on CA (including CA1, CA2 and CA3), which were in line with previous works. CA1 is the main output structure of the hippocampal cortex, and may serve to transfer information to cortical and subcortical structures (Jones and McHugh, 2011). As an area between CA3 and CA1, CA2 has extensive connectivity with intra- and extra- hippocampal cortex, and plays an important part in cognitive and emotional processes (Chevaleyre and Piskorowski, 2016; Hitti and Siegelbaum, 2014).

The results of whole brain-to-seeds Granger causality analysis showed that in comparison to the control condition, left thalamus activation preceded increased activation in CA, DG and Subc activation, and the activation increase in right thalamus predicted subsequent increases in activation of DG after stress exposure. These findings are consistent with previous studies showing that the thalamus has dense anatomical connectivity with the hippocampus (Aggleton et al., 2010; Tsanov et al., 2011). The thalamus is believed to be a gateway for primary sensory output to the cerebral cortex and is involved in various cognitive functions, such as attention, consciousness and emotion (J. Chang and Yu, 2018b; Crick and Koch, 2003; Haber and Calzavara, 2009; Shipp, 2004; Ward, 2013). Previous studies found that the thalamus played a part in creating a wide range of emotions, especially negative emotions, and was involved in emotion memory storage through its connection with the hippocampus (Lane et al., 1997; Ward, 2013). In the current study, the increased thalamus-hippocampus coupling under stress might be related to the formation and consolidation of emotional memory induced by TSST. In line with this speculation, previous animal and human studies found that the connectivity between the thalamus and hippocampus was critical for episodic memory formation, and a lesion of the anterior thalamus could influence performance on spatial memory tasks (Tsanov, 2015; van Groen et al., 2002; Wilton et al., 2001).

Importantly, the SVM results showed that the combination of Granger causality values from CA to left insula, from CA to left midbrain, from DG to left midbrain, and from right thalamus to DG predicted the stress condition with high accuracy of 76.7% (*p* = 0.001 after the permutation test). These results suggest that the information flows in the thalamus-hippocampus-insula/midbrain circuit could be used as reliable biomarkers to distinguish these two psychological states, further supporting the crucial role of this circuit in the processing of stress. As far as we have known, this is the first study using a machine learning method to differentiate between the acute stress state and the control state. Previous studies have adopted the machine learning algorithm to find the features (e.g., the functional connectivity between brain regions) with high discriminative power for group classification and use them to assist the diagnosis of disease (Anderson and Cohen, 2013; Li et al., 2018). Our findings may provide biomarkers in the diagnosis of stress-related disorders and contribute to precise intervention.

It is worth noting that there are some limitations in our study. Firstly, because BOLD signal is an indirect reflection of neuronal activity, and hemodynamic responses are various in different brain regions, applying GCA to fMRI data has sparked a great deal of controversy (O. David et al., 2008; K. Friston, Moran and Seth, 2013; Wen et al., 2013). Interestingly, a previous study found that granger prediction is robust to various hemodynamic responses but is vulnerable to down sampling and data noise (Seth et al., 2013). However, there are also some stimulation studies showing relatively high robustness of granger prediction in fMRI data analysis (Deshpande et al., 2010a, Deshpande et al., 2010b; Schippers et al., 2011). Thus further studies are needed to understand this method better. In addition, unlike dynamic causal modeling, GCA just describes the information flow of fMRI data but cannot explore the underlying physical-causal mechanisms through statistical models (Cohen, 2014; Seth et al., 2015). Graph theory is widely used to explore the structural and functional brain networks underlying various cognitive functions, with brain regions as nodes and connectivity among them as edges (Bullmore and Sporns, 2009; Harvy et al., 2019; Li et al., 2019). Further, the edges could be undirectional (e.g., correlation) or directional (e.g., Granger causality) (Bullmore and Sporns, 2009). Compared with GCA, graph theory could provide more macroscopical information about how the brain is coordinated (Zhan and Yu, 2015). Future studies may use graph theoretical analysis to delineate the dynamic brain network changes under stress holistically. Secondly, previous studies showed that stress enhances the functional connectivity between amygdala and hippocampus (Kiem et al., 2013; Vaisvaser et al., 2013). We did not find a similar significant alteration of the effective connectivity between hippocampus and amygdala under stress. This might be due to the fact that the Granger causal analysis we conducted estimates directional connectivity rather than undirectional functional connectivity. Further, unlike previous studies which only studied the hippocampus as a whole, we focused on the effects of stress on effective connectivity from and to different hippocampal subregions. Thirdly, due to the low spatial resolution of functional imaging, the hippocampal ROI may reflect the signal from adjacent regions. Our exploratory results need to be interpreted without caution. We used DARTEL to create an average structural brain template from all subjects' T1 images and registered BOLD volumes to a MNI template using the DARTEL template, which could increase the precision of registration. High resolution anatomical and functional images collected using 7 T MRI scanner may also be used to study hippocampal subregions (Berron et al., 2017). Fourthly, no autonomic nervous system responses were recorded in our study. The autonomic nervous system (ANS) and HPA axis are the main systems to maintain homeostasis under stress (Ulrich-Lai and Herman, 2009). Previous studies found that the HPA axis was more sensitive to social stressors (e.g. the TSST), while the ANS was associated with the rapid alteration of physiological states induced by physical stressors, such as cold stressor (McRae et al., 2006; Ulrich-Lai and Herman, 2009). Future studies are needed to explore the relationships between changes in ANS and hippocampus's effective connectivity patterns and how different types of stressors influence them. Finally, participants in the current study were young Chinese adults in the age range of 18–25, when brain development is still ongoing. It remains to be tested whether our findings can be extended to adults with fully mature brain development. Given the profound cultural differences in how people cope with stressor (Palsane and Lam, 1996; Taylor et al., 2007), individuals from western cultures may respond differently to the TSST and show distinct neural patterns (Allen et al., 2014). A culture social neuroscience approach to study stress may shed lights on the cultural variation in psychological and neural processes under stress.

The present study is the first study combining Granger causal analysis and a support vector machine to explore the alterations of effective connectivity from and to hippocampal subregions after stress, and to detect the effective connectivity that distinguishes stress and control brain states. The findings showed that stress altered the effective connectivity in the thalamus-hippocampus-insula/midbrain circuit, and the changes in this circuit could predict the brain state at subject level with high accuracy. These brain structures and the interrelations among them are documented to be involved in emotional information processing and storage, and habitual responses (Egorova et al., 2015; D. Mobbs et al., 2009; D Rangaprakash et al., 2017a, Rangaprakash et al., 2017b; Tsukiura et al., 2012; Ward, 2013). We speculate that the circuit might be related to the encoding of salient negative information after acute social stress in order for the healthy individual to better cope with similar future stressful events. Our study could be further helpful to explain the neural mechanisms underlying emotion dysregulation symptoms in stress-related psychiatric disorders, such as MDD and PTSD. Previous studies have largely focused on the hippocampus as a unitary structure (Dunkley et al., 2014; Logue et al., 2018). There is accumulating evidence to support the differential roles of the hippocampal subregions in PTSD symptoms and associated memory processes (Malivoire et al., 2018). Given the different structural and functional connectivities of hippocampal subregions, investigating hippocampal rsFC at a subregional level in the present study may help elucidate the functional neurobiology of PTSD.

## Declaration of conflicting interests

The authors declared that they had no conflicts of interest with respect to their research, authorship or the publication of this article.

## Author contributions

R. Yu developed the study concept. J. Chang analyzed and interpreted the data under the supervision of R. Yu. J. Chang drafted the manuscript, and R. Yu provided critical revisions. All authors approved the final version of the manuscript for submission. We thank J. Hu for collecting the data.
